# Adjustable multicolor up-energy conversion in light-luminesce in Tb^3+^/Tm^3+^/Yb^3+^ co-doped oxyfluorifFde glass-ceramics containing Ba_2_LaF_7_ nanocrystals

**DOI:** 10.1038/s41598-017-05943-4

**Published:** 2017-07-26

**Authors:** Zhencai Li, Dacheng Zhou, Yong Yang, Peng Ren, Jianbei Qiu

**Affiliations:** 1 Faculty of Material Science and Engineering, Kunming University of Science and Technology, Kunming, 650093 China; 2 Key Lab. of Advanced Materials of Yunnan Province, Kunming, 650093 China

## Abstract

Transparent oxyfluoride glasses with highly efficient up-energy conversion (UEC) luminescence were developed in the 45SiO_2_-15Al_2_O_3_-12Na_2_CO_3_-21BaF_2_-7LaF_3_-xTbF_3_-yTmF_3_-zYbF_3_ composition (in mol%), and structural investigation by X-ray diffraction (XRD) and transmission electron microscopy (TEM) confirmed the formation of face-centered cubic Ba_2_LaF_7_ nanocrystals. The colors of UEC luminescences could be tuned easily by adjusting the concentration of doped rare earth ions and the excitation power of laser simultaneously. The relationship between the emission intensity of Tb^3+^/Tm^3+^/Yb^3+^ co-doped oxyfluoride glass-ceramics and the excitation pump power revealed that three-photon and two-photon absorptions predominated in the conversion process from the infrared into blue and red luminescences, respectively. A novel UEC mechanism of red emission from Tm^3+^ was proposed, energy transfers from Yb^3+^ to Tm^3+^ and Tb^3+^ and from Tm^3+^ to Tb^3+^ were evidenced. The possible mechanism responsible for the color variation of UEC in Tb^3+^/Tm^3+^/Yb^3+^ co-doped was discussed.

## Introduction

In recent years, increasing attention has been paid to the generation of white light sources for a variety of application purposes, such as white light emitting diodes (W-LEDs), back lighting, solid-state multicolor three-dimensional displays and so on. One of the effective ways for generating white light is to use rare earth (RE) ions doped material based on frequency up-energy conversion (UEC) process, which can convert low energy near-infrared radiation (NIR) into high energy visible radiation via multiphoton processes^1–3^. Lanthanide ions are suitable candidates for the UEC process owing to their abundant energy levels and narrow emission spectral lines^4–7^. However, their applications are greatly restricted due to the poor chemical stability and low damage threshold of the host materials that are limited to films and phosphors. Compared with abovementioned host materials, oxyfluoride glass-ceramics materials have also attracted great concerns since the first laser demonstration on Nd:YAG ceramic in 1995^8^. Oxyfluoride glass-ceramics can be highly transparent in the ultraviolet–visible–infrared range, which is beneficial to the output of UEC luminescences. Above all, oxyfluoride glass-ceramics combine the low phonon energy of fluoride crystals and the high chemical and mechanical stability of oxide glasses^9–12^, and thus ensure their convenient use in devices such as lasers and sensors.

In order to achieve multicolor visible light in lanthanide doped glass-ceramics^13, 14^, the luminescence and relative intensity control of the three primary colors of red, green and blue (RGB) is required^15, 16^. In addition, it is necessary to develop a novel method to produce multicolor visible light. Indeed, there have been some reports on the control of luminescences in three primary colors through the UEC method. For example, Downing *et al*.^17, 18^. reported simultaneous generation of RGB fluorescences from fluoride glasses triply doped with Tm^3+^, Er^3+^ and Pr^3+^ using three different pairs of near-infrared laser excitation sources. Despite the fact that multiple-pump wavelength configuration has been produced, a single-pump scheme is still in great need. Recently, the reports on Tm^3+^/Er^3+^/Yb^3+^ ions doped glass-ceramics have been used to realize white light emission and color tunability by adjusting pump power or Ln^3+^ concentration via the UEC process^19–21^. As to our best knowledge, there is little attention paid to the UEC excitation of luminescence materials to produce controllable colors through multiple ways simultaneously under a single-pump scheme.

In the present study, the multicolor tunability through the UEC process by optimizing the concentrations of Tm^3+^, Tb^3+^ and Yb^3+^ ions in Ba_2_LaF_7_ nanocrystals was reported for the first time. The UEC efficiency was dramatically improved by the addition of Yb^3+^ ions, Tb^3+^ ions and Tm^3+^ ions. The UEC white light could be obtained from the combination of green component of Tb^3+^ ions, blue and red components of Tm^3+^ ions, with Yb^3+^ ions as sensitizers in the UEC process. By adjusting the concentration of Yb^3+^, Tb^3+^ and Tm^3+^ ions or the excitation power of laser, a wide color adjustability was achieved, and the RGB color tunability as a function of pump power from 0.26 to 1.65 W using 980 nm laser excitation was discussed. Meanwhile, the color coordinates were evaluated and the possibility to obtain white light from the prepared samples was analyzed using CIE 1931 chromaticity diagram. In addition, the possible UEC mechanisms for energy transfer processes between Tb^3+^, Tm^3+^ and Yb^3+^ ions were also discussed.

## Experimental

Reagents of SiO_2_ (99.99%), Al_2_O_3_ (99.99%), Na_2_CO_3_ (99.99%), BaF_2_ (99.99%), LaF_3_ (99.99%), TbF_3_ (99.99%), TmF_3_ (99.99%) and YbF_3_ (99.99%) were used as raw materials. Precursor glass samples (about 10 g) were prepared respectively with the following molar compositions: 45SiO_2_-15Al_2_O_3_-12Na_2_CO_3_-21BaF_2_-7LaF_3_-0.5TbF_3_-0.01TmF_3_-zYbF_3_ (z = 1, 2, 3 and 4 in mol%, which were named as SABYb-1, SABYb-2, SABYb-3 and SABYb-4 respectively), 45SiO_2_-15Al_2_O_3_-12Na_2_CO_3_-21BaF_2_-7LaF_3_-0.5TbF_3_-yTmF_3_-4YbF_3_ (y = 0.005, 0.015, 0.025 and 0.04 in mol%, which were named as SABTm-1, SABTm-2, SABTm-3 and SABTm-4 respectively), and 45SiO_2_-15Al_2_O_3_-12Na_2_CO_3_-21BaF_2_-7LaF_3_ -xTbF_3_-0.015TmF_3_-4YbF_3_ (x = 0.1, 0.2, 0.4 and 0.6 in mol%, which were named as SABTb-1, SABTb-2, SABTb-3 and SABTb-4 respectively). For each batch, the raw materials were fully mixed and melted in a covered Alumina crucible in air atmosphere at 1400 °C for 45 min, and then cast into a brass mold, where the sample was slowly cooled down to room temperature. All the glasses were annealed at 500 °C for 8 h to remove thermal strains. The samples were cut into cuboids with the dimensions of 10 mm × 10 mm × 2 mm and then polished for optical measurements. All measurements were performed at ambient temperature.

Differential thermal analysis (DTA) thermograms were measured in a nitrogen atmosphere on STA-449F3 (NETZSCH). To identify the phase composition of the samples, XRD analysis was carried out with a powder diffractometer using Cu Kα radiation. The sizes, shapes, structures and component compositions of the as-prepared nanocrystals were characterized by Scanning electron microscopy (SEM, QUANTA 200) and transmission electron microscopy (TEM, JEM-2100) at a voltage of 30 K and 200 KV. The UEC emission spectra of Tb^3+^/Tm^3+^/Yb^3+^ co-doped glass-ceramics in the wavelength range from 425 to 725 nm were recorded with a HITACHI F-7000 fluorescence spectrophotometer under the 980 nm laser diode excitation. The polished SABYb-2 glass sample was selected for heat treatment at four different temperatures of 600 °C, 610 °C, 620 °C and 640 °C for 2 h respectively to form transparent glass-ceramics, and the glass-ceramics were named as SABYb-2-600 SABYb-2-610, SABYb-2-620 and SABYb-2-640 respectively.

## Results and Discussion

**Figure 1:**
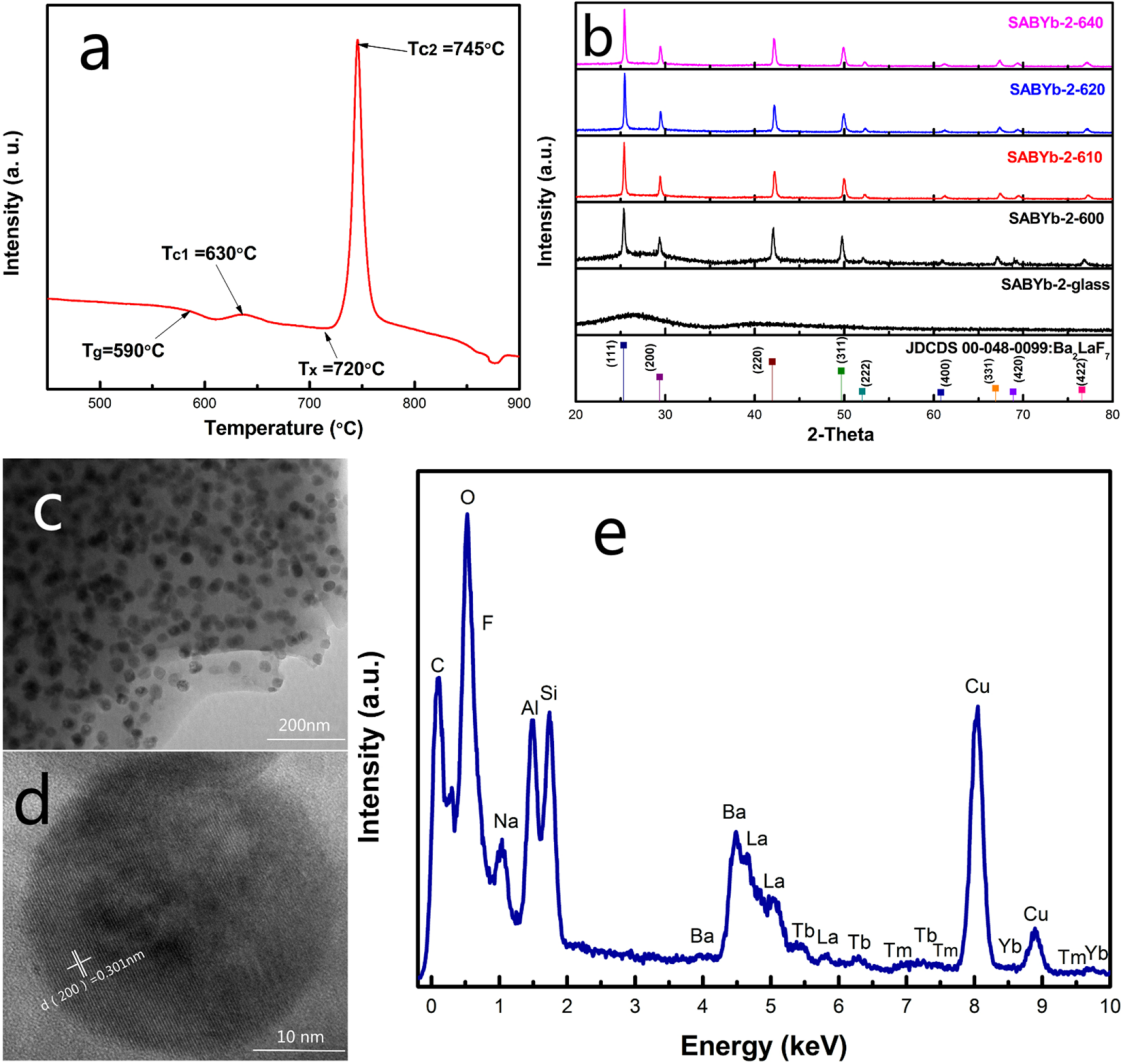
(**a**) The DTA curve of the SABYb-2 glass sample. (**b**) XRD patterns of the SABYb-2 glass and glass-ceramics after heat treatment at 600 °C, 610 °C, 620 °C and 640 °C for 2 h. (**c**) TEM micrograph of SABYb-2 glass-ceramics after heat-treated at 640 °C for 2 h. (**d**) High resolution transmission electron microscope (HRTEM) image of SABYb-2 glass-ceramics. (**e**) EDX spectra from an individual Ba_2_LaF_7_ nanocrystal.

Figure 1(a) shows the DTA curves of the SABYb-2 glass. It can be seen that the transition temperature (T_g_) of the glass is located at 590 °C. There is a crystallization peak at the temperature of T_c1_ = 630 °C, and an obvious crystallization peak appears at the temperature of T_c2_ = 745 °C (with the crystallization onset temperature of T_x_ = 720 °C). The temperature difference ΔT between T_x_ and T_g_ (ΔT = T_x_−T_g_) is generally used as a rough indicator of glass thermal stability. Here, ΔT = 130 °C > 100 °C, indicating that the prepared glass is stable and suitable for applications such as fiber amplifiers and lasers^22, 23^. Therefore, according to DTA results, transparent glass ceramics can be prepared by heat treatment at the crystallization peak near 630 °C by appropriately controlling the crystallization temperature and process.

Figure 1(b) shows the XRD patterns of SABYb-2 glass and its glass-ceramics nanocrystals after heat treatment at different temperatures of 600 °C, 610 °C, 620 °C and 640 °C for 2 h. The crystalline grain size D for a given (*hkl*) plane can be estimated from the XRD patterns following the Scherrer equation:

1 $$D=\frac{{\rm{K}}\lambda }{\beta \,\cos \,\theta }$$

where K = 0.89, λ is the wavelength of the incident XRD (for Cu Kα, λ = 0.154056 nm), β represents the corrected half width of diffraction peak and θ is the Bragg angle of X-ray diffraction peak^24^. By using the Scherrer equation, the average grain sizes of Ba_2_LaF_7_ nanocrystals can be calculated to be about 12 nm, 18 nm, 26 nm and 39 nm for SABYb-2-600, SABYb-2-610, SABYb-2-620 and SABYb-2-640 samples, respectively. It is obvious that the average grain size of Ba_2_LaF_7_ nanocrystals increases with increasing temperature of heat treatment from 600 to 640 °C.

Figure 1(c) gives the TEM image of the SABYb-2-640 glass-ceramics. It can be seen that Ba_2_LaF_7_ nanocrystals distribute uniformly in the glass matrix (Fig. 2(c and d) in Supplementary Information). Their average grain size is about 39 nm, which is similar to that calculated by the Scherrer equation. The high resolution transmission electron microscopy (HRTEM) image with the d-spacing structure is shown in Fig. 1(d), and the d-spacing value of (200) plane is determined as 0.301 nm. Figure 1(e) provides the compositional analysis results of an individual Ba_2_LaF_7_ nanocrystal grain measured by an energy dispersive X-ray detector (EDX). As can be seen, the peaks of Tb, Tm and Yb elements all appear on the spectrum curve, demonstrating that Tb^3+^, Tm^3+^ and Yb^3+^ ions have been effectively embedded into the Ba_2_LaF_7_ host lattice^25^.

**Figure 2:**
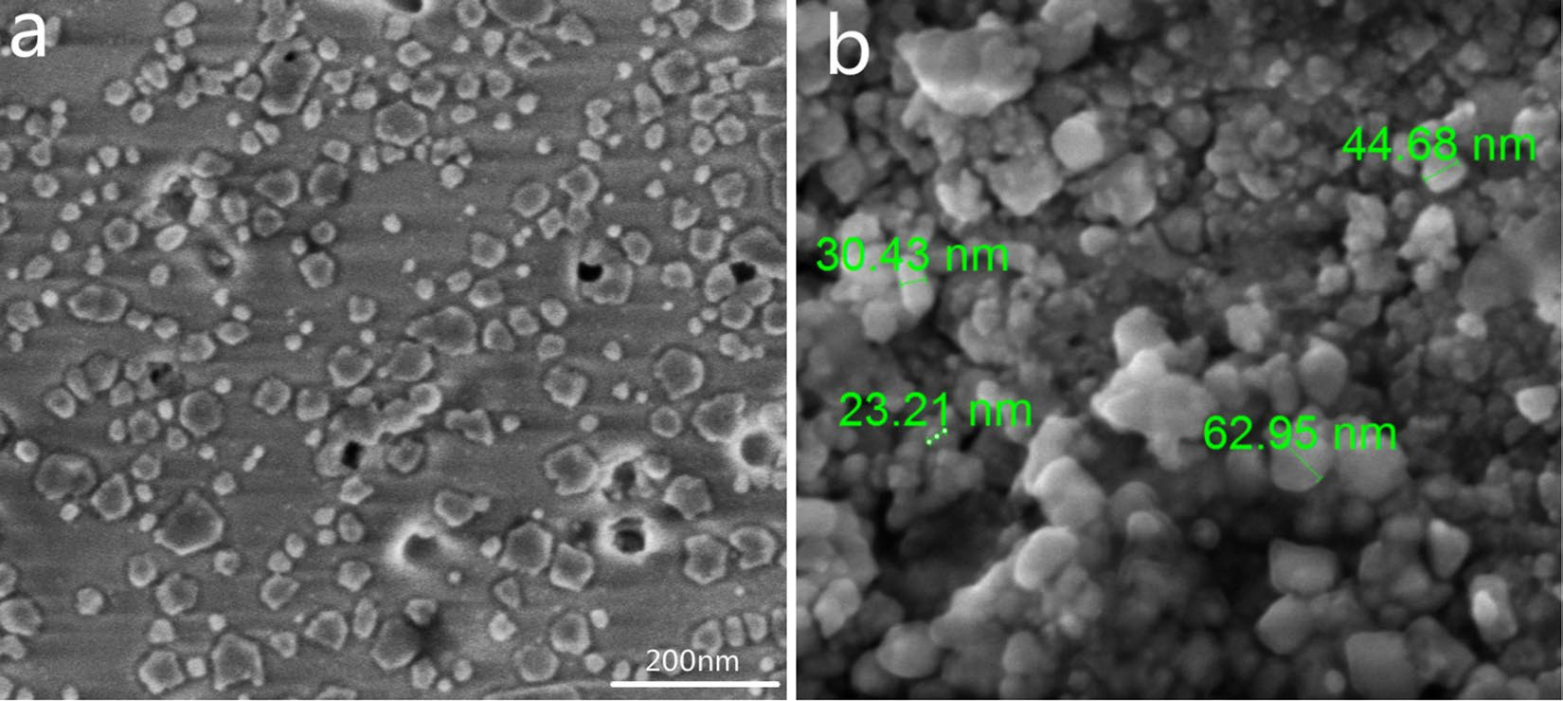
The SEM images of the SABYb-2-640 glass-ceramics after heat-treated at 640 °C for 2 h.

Figure 2 shows the SEM images of the SABYb-2-640 glass-ceramics. In Fig. 2(a), the light areas represent crystalline regions enriched in atoms with high atomic number (rare earth elements and barium) and the dark areas represent the glass matrix mostly containing lighter atoms including sodium, aluminum and silicon. It can be seen that crystalline regions are uniformly distributed in the glassy matrix for all glass-ceramics, which indicates a homogenous crystallization process. In most oxyfluoride glass-ceramics, the diffusion barrier containing glass formers surrounds the fluoride nanocrystals and prevents their further growth. As a result, small Ba_2_LaF_7_ nanocrystals can precipitate in the glassy matrix, and single crystals (see Fig. 2(b)) can be obtained, indicating a relatively low viscosity of the base glass at the crystallization temperature^26^. Although the nanoparticles are not exactly of the same size, they have similar grain size with an average value of about 39 nm. In addition, the glass sample was synthesized by conventional quenching technique after the late heat treatment crystallization process. It should be noticed that the uniformity of the nanoparticles synthesized by this technique is worse than that of the nanoparticles synthesized by the hydrothermal method. Hence, the SEM images of Ba_2_LaF_7_ will be more helpful for the nanostructures.

**Figure 3:**
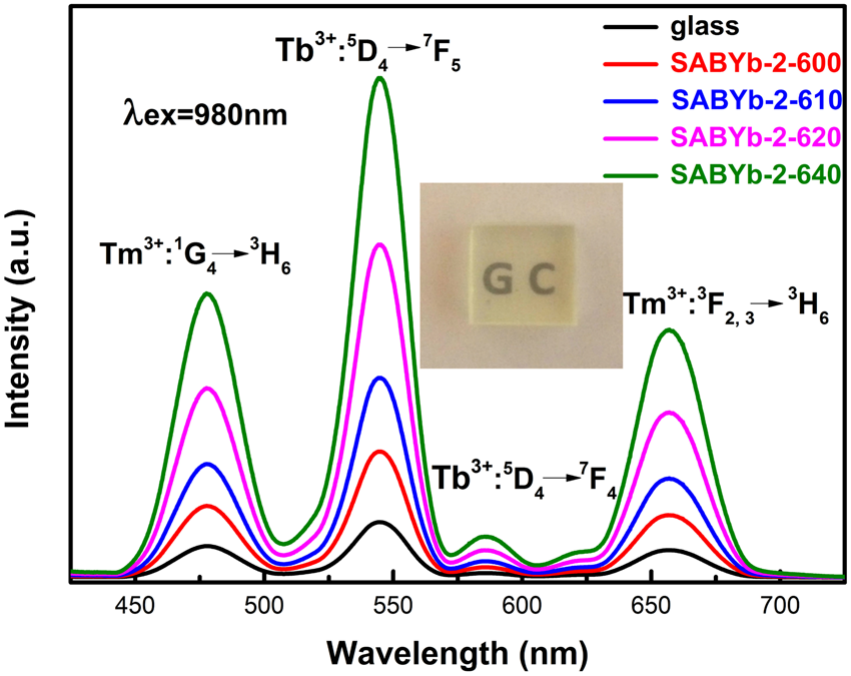
The UEC emission spectra of SABYb-2 glass and glass-ceramics, which were heat-treated at 600 °C, 610 °C, 620 °C and 640 °C for 2 h.

Figure 3 shows the UEC emission spectra of the SABYb-2 glass and glass-ceramics. Here, we compare the intensities of emission peaks at visible wavelengths of 476 nm (Tm: ^1^G_4_ → ^3^H_6_), 546 nm (Tb: ^5^D_4_ → ^7^F_5_), 584 nm (Tb: ^5^D_4_ → ^7^F_4_) and 657 nm (Tm: ^3^F_2,3_ → ^3^H_6_) respectively. The intensity of visible transitions confirms that light scattering is not dominant in the material till heat treatment up to 640 °C. In the annealing process from 600 to 640 °C, the average grain size increases from 12 to nearly 39 nm, as shown in Table 1. The corresponding XRD patterns confirm their good crystallinity and the optical image of glass-ceramics sample displays good transparency in the visible wavelength range (see inset of Fig. 3). On this basis, it can be deduced that the spectral enhancement in the glass-ceramic materials is predominantly attributed to the presence of Ba_2_LaF_7_ crystals that form above the glass transition temperature (T_g_ = 590 °C) and dominate the phase composition up to 640 °C^27^. From the Ba_2_LaF_7_ structure, which nucleates and grows between 600 °C and 640 °C, it is known that RE ions are basically dispersive into Ba_2_LaF_7_ nanocrystals of the glass-ceramics^28^. As a result, the distance between RE ions becomes closer, which results in the enhancement of UEC luminescences.

**Table 1:** The UEC emission intensity of 546 nm and crystalline grain size of SABYb-2 glass-ceramics heat-treated at temperatures of 600 °C, 610 °C, 620 °C and 640 °C for 2 h.

Temperature (°C)	UEC emission intensity of 546 nm	Crystalline grain size (nm)
600	77	12
610	92	18
620	108	26
640	121	39

The UEC mechanism mainly focuses on forward energy transfer. However, this may be a very biased view, since both the phonon energy and lifetimes during the UEC process are comparable to those of lower phonon energy glass hosts where both forward and backward energy transfer processes have been recorded and characterized. Recently, Arai *et al*.^29^ doped fluorophosphate glass with Yb^3+^ and Tb^3+^ ions as high as 20 mol%, and reported an energy transfer efficiency up to 30% from Yb^3+^ to Tb^3+^ via the UEC process. On the other hand, we also confirmed that there were other energy transfer mechanisms that suppressed the UEC process, i.e., the backward energy transfer process, such as phonon assisted energy transfer, cooperative cross relaxation and so on refs 30, 31. Although the energy transfer efficiency is sufficiently high in fluorophosphate glass, the backward energy transfer process is not negligible, so that it is very likely for the occurrence of backward energy transfer from Tb^3+^ to Yb^3+^, which decreases the intensity of the observed green emission at 546 nm. Based on the above, we have added experimental parts about forward and backward energy transfer processes. The Table 2 show the added experimental parts, the glass samples were heat-treated at 640 °C. The cooperative energy transfer rate (W_CET_) and the transfer efficiency (η_CET_) from Yb^3+^ to Tb^3+^ can be quantified using the following expressions^31, 32^:

2 $${{\rm{W}}}_{{\rm{CET}}}=\frac{1}{{{\rm{\tau }}}_{{\rm{Yb}}-{\rm{Tb}}}}-\frac{1}{{{\rm{\tau }}}_{{\rm{Yb}}}}$$

3 $${{\rm{\eta }}}_{{\rm{CET}}}=\frac{{{\rm{W}}}_{{\rm{CET}}}}{1/{{\rm{\tau }}}_{{\rm{Yb}}-{\rm{Tb}}}}$$

where τ_Yb−Tb_ is the lifetime of the Yb^3+^: ^2^F_5/2_ level in the Yb^3+^−Tb^3+^ co-doped sample (sample 1), and τ_Yb_ is the lifetime of the Yb^3+^: ^2^F_5/2_ in the Yb^3+^ doped sample (sample 2). Accordingly, the following formulas can be used to quantify the backward energy transfer rate (W_BT_) and the corresponding transfer efficiency (η_BT_):

4 $${{\rm{W}}}_{{\rm{BT}}}=\frac{1}{{{\rm{\tau }}}_{{\rm{Tb}}-{\rm{Yb}}}}-\frac{1}{{{\rm{\tau }}}_{{\rm{Tb}}}}$$

5 $${{\rm{\eta }}}_{{\rm{BT}}}=\frac{{{\rm{W}}}_{{\rm{BT}}}}{1/{{\rm{\tau }}}_{{\rm{Tb}}-{\rm{Yb}}}}$$

where τ_Tb−Yb_ is the lifetime of the Tb^3+^: ^5^D_4_ level in the Yb^3+^−Tb^3+^ co-doped sample (sample 1) and τ_Tb_ is the lifetime of the Tb^3+^: ^5^D_4_ in the Tb^3+^ doped sample (sample 3).

**Table 2:** Compositions of the samples doped with Yb^3+^ or Tb^3+^ ions.

Type	Host composition (mol%)	Doping scheme (mol%)
Sample 1	45SiO_2_-15Al_2_O_3_-12Na_2_CO_3_-21BaF_2_-7LaF_3_	0.5TbF_3_-1YbF_3_
Sample 2	45SiO_2_-15Al_2_O_3_-12Na_2_CO_3_-21BaF_2_-7LaF_3_	1YbF_3_
Sample 3	45SiO_2_-15Al_2_O_3_-12Na_2_CO_3_-21BaF_2_-7LaF_3_	0.5TbF_3_

Tables 3 and 4 show the forward and backward energy transfer efficiencies for sample 1. These quantitative results have been calculated by substituting the lifetimes measured in samples 1, 2 and 3 into Eqs (2)–(5). As shown in Tables 3 and 4, the forward energy transfer efficiency is considerably higher than the backward energy transfer efficiency. Based on the above results, in the next work, because the rare earth ion doping amount is different, so we mainly study the forward energy transfer.

**Table 3:** Forward energy transfer data (2Yb^3+^: ^2^F_5/2_ → Tb^3+^: ^5^D_4_).

τ_Yb−Tb_	τ_Yb_	W_CET_	η_CET_
1.362 ms	1.63 ms	92.73 s^−1^	12.58%

**Table 4:** Backward energy transfer data (Tb^3+^: ^5^D_4_ → 2Yb^3+^: ^2^F_5/2_).

τ_Yb−Tb_	τ_Yb_	W_BT_	η_BT_
1.535 ms	1.541 ms	2.54 s^−1^	0.39%

**Figure 4:**
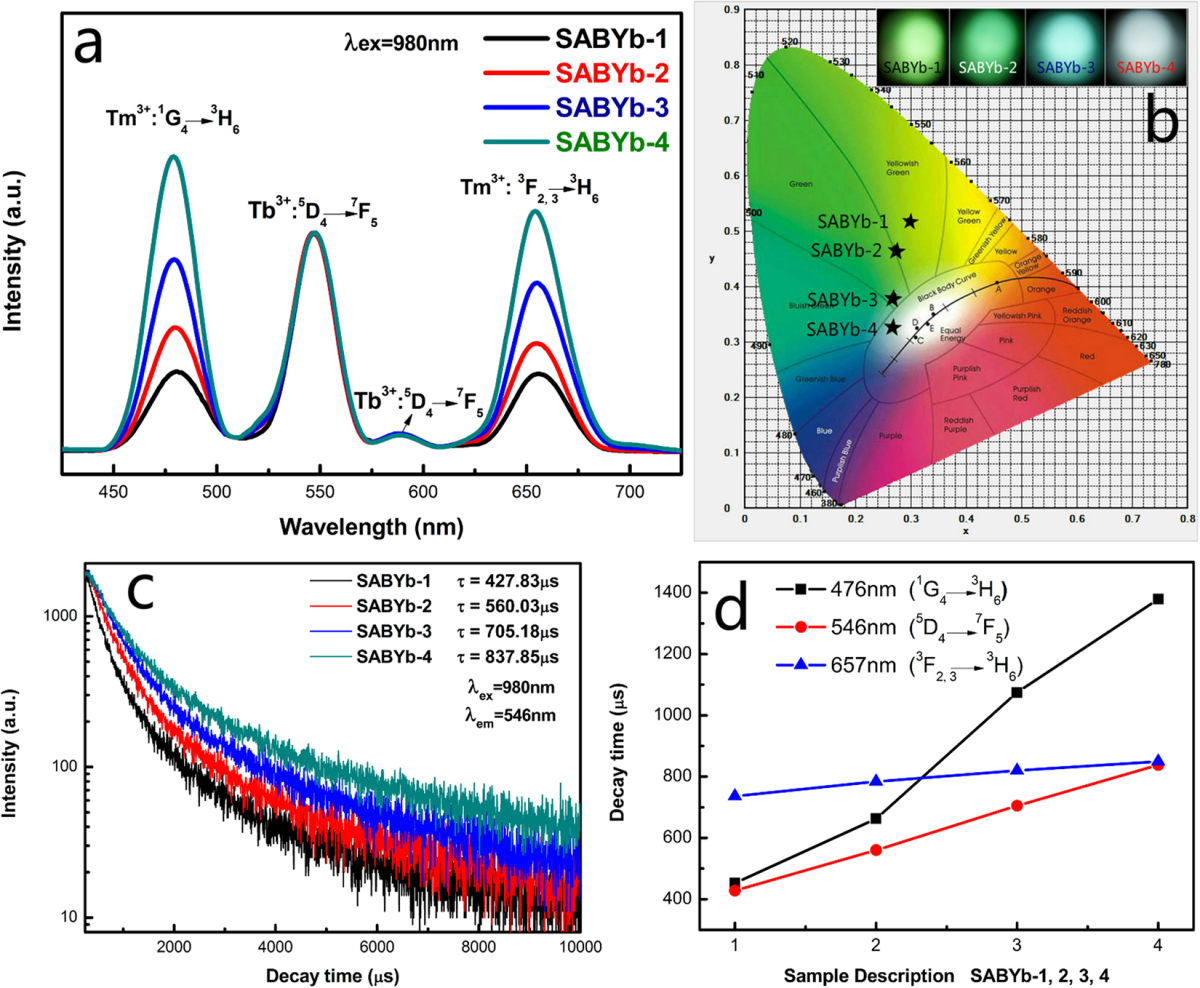
(**a**) The UEC emission spectra of SABYb-1, SABYb-2, SABYb-3 and SABYb-4 glass-ceramics. (**b**) CIE (X, Y) coordinate diagram showing chromaticity points of Tb^3+^ and Tm^3+^ luminescence in the nanocomposites. (**c**) Fluorescence decay curves of Tb^3+^ ions at 546 nm in the SABYb glass-ceramics. (**d**) Decay behavior of ^5^D_4_, ^1^G_4_ and ^3^F_2,3_ levels for the SABYb glass-ceramics under 980 nm excitation.

Figure 4 shows the UEC emission spectra and decay curves of SABYb-1, SABYb-2, SABYb-3 and SABYb-4 glass-ceramics heat-treated at 640 °C. Four visible UEC emission bands from 425 to 725 nm can be observed in Fig. 4(a). In comparison with those in Fig. 4(a), the UEC emission intensities at 476 nm (Tm^3+^: ^1^G_4_ → ^3^H_6_), 546 nm (Tb^3+^: ^5^D_4_ → ^7^F_5_), 584 nm (Tb^3+^: ^5^D_4_ → ^7^F_4_) and 657 nm (Tm^3+^: ^3^F_2,3_ → ^3^H_6_) are all enhanced dramatically with increasing concentration of Yb^3+^ ions in the SABYb glass-ceramics. The population of Tb^3+^ ions in the ^5^D_4_ excited state level is thought to be produced through the cooperative energy transfer (CET) process among a pair of Yb^3+^ donor ions and a Tb^3+^ acceptor ion, which can be expressed as follows^33, 34^: ^2^F_5/2_ (Yb^3+^) + ^2^F_5/2_ (Yb^3+^) + ^7^F_6_ (Tb^3+^) → ^5^D_4_ (Tb^3+^) + ^2^F_7/2_ (Yb^3+^) + ^2^F_7/2_ (Yb^3+^). Meanwhile, Yb^3+^ energy transfer to the ^1^G_4_ excited state level of Tm^3+^ ions and decay radiation to the ^3^H_6_ ground state also generate the intense blue emission at around 476 nm. The major contribution to the red emission at around 657 nm is attributed to the ^3^F_2,3_ → ^3^H_6_ transition^35^. The emission spectra of SABYb-1, SABYb-2, SABYb-3 and SABYb-4 glass-ceramics can be easily converted to the Commission Internationale de L’Eclairage (CIE) chromaticity diagram, as plotted in Fig. 4(b). The luminescence color changes from yellowish green (SABYb-1), to green (SABYb-2), then to bluish green (SABYb-3), and finally to white (SABYb-4)^36^. Figure 4(c) illustrates the decay time of ^5^D_4_ (Tb^3+^) energy level with increasing concentration of Yb^3+^ ions. Here, only approximate single-exponential luminescence decay curves can be obtained. Hence, the lifetimes characterized by decay lifetime τ can be deduced by the following formula:

6 $${\rm{\tau }}=\frac{{\int }^{}{\rm{tI}}({\rm{t}}){\rm{dt}}}{{\int }^{}{\rm{I}}({\rm{t}}){\rm{dt}}}$$

The average lifetimes of ^5^D_4_ state were determined as 427.83 μs, 560.03 μs, 705.18 μs and 837.85 μs. Figure 4(d) shows the variation of decay times of ^1^G_4_ (Tm^3+^), ^5^D_4_ (Tb^3+^) and ^3^F_2,3_ (Tm^3+^) energy levels with increasing concentration of Yb^3+^ ions. It can be seen that the energy transfers from Yb^3+^ to Tb^3+^ and Tm^3+^ are gradually strengthened. That is to say, by adjusting the concentration of Yb^3+^ ions, the adjustable multicolor and UEC near-white light emitting of the SABYb glass can be achieved in this experiment.

**Figure 5:**
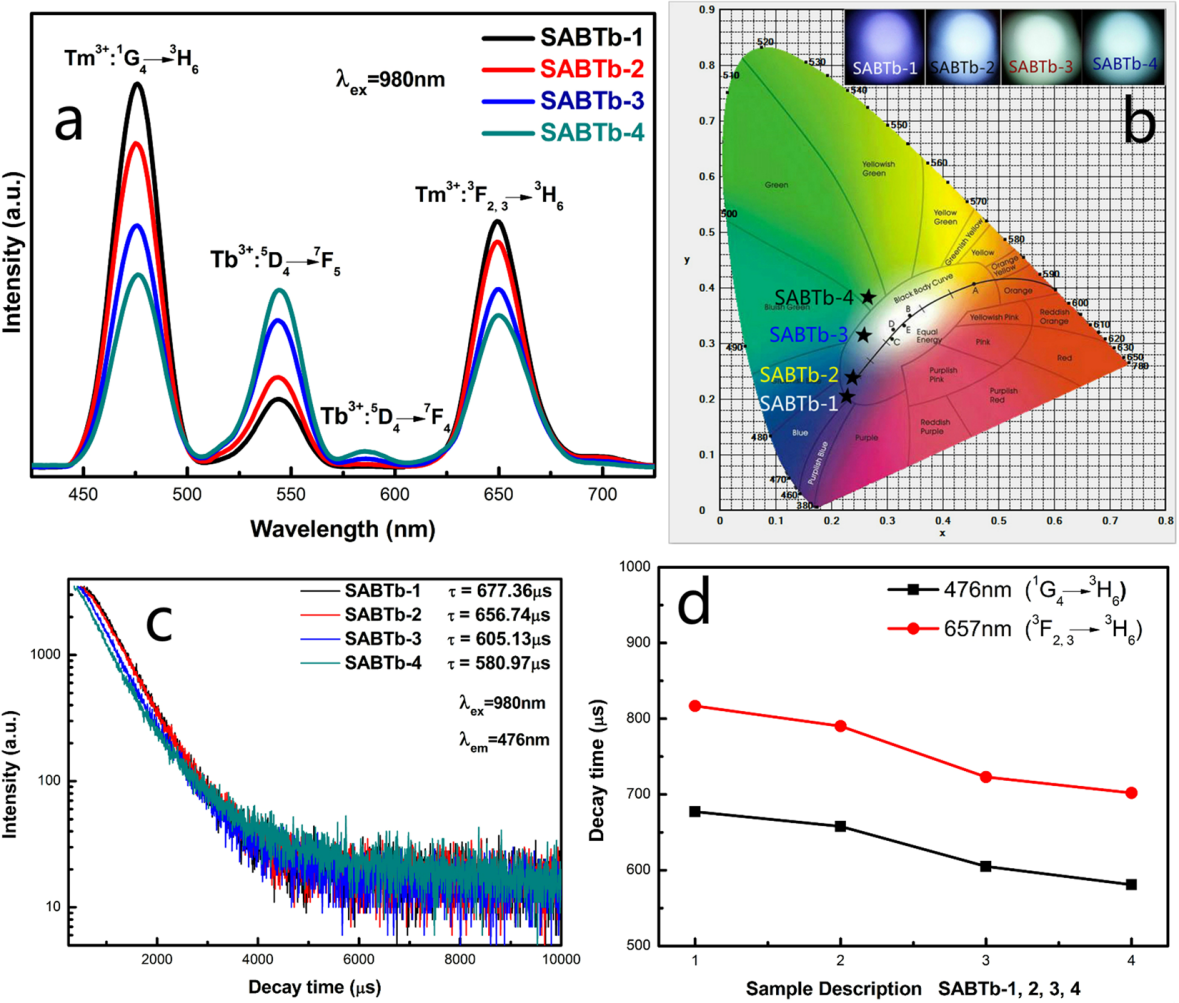
(**a**) The UEC emission spectra of SABTb-1, SABTb-2, SABTb-3 and SABTb-4 glass-ceramics under 980 nm excitation. (**b**) CIE (X, Y) coordinate diagram showing chromaticity points of Tb^3+^ and Tm^3+^ luminescence in the glass-ceramics. (**c**) Fluorescence decay curves of Tm^3+^ ions at 476 nm in the SABTb glass-ceramics under 980 nm excitation. (**d**) Decay behavior of ^5^D_4_, ^1^G_4_ and ^3^F_2,3_ levels for the SABTb glass-ceramics.

Figure 5 shows the UEC emission spectra and decay times of SABTb-1, SABTb-2, SABTb-3 and SABTb-4 glass-ceramics heat-treated at 640 °C. As can be seen from Fig. 5(a), with increasing concentration of Tb^3+^ ions in the SABTb glass-ceramics, the UEC emission bands at 546 nm (Tb^3+^: ^5^D_4_ → ^7^F_5_) and 584 nm (Tb^3+^: ^5^D_4_ → ^7^F_4_) are enhanced dramatically whereas those at around 476 nm (Tm^3+^: ^1^G_4_ → ^3^H_6_) and 657 nm (Tm^3+^: ^3^F_2,3_ → ^3^H_6_) originated from Tm^3+^ ions are gradually weakened. This phenomenon can be explained from three aspects. Firstly, the increasing concentration of Tb^3+^ ions can lead to the increase of luminescent centers, thus enhancing the emission intensity at 546 nm (Tb^3+^: ^5^D_4_ → ^7^F_5_). Secondly, the gradual increase of Tb^3+^ ions surrounded by Tm^3+^ ions can hinder the energy transfer from Yb^3+^ to Tm^3+^, resulting in the decrease of Tm^3+^ emission. Thirdly, the possible energy transfer from Tm^3+^ to Tb^3+^ ions contributes to the emission intensity at 546 nm. The mechanism of energy transfer from Tm^3+^ to Tb^3+^ ions is proposed as follows: ^1^G_4_ (Tm^3+^) + ^7^F_5_ (Tb^3+^) → ^5^D_4_ (Tb^3+^) + ^3^H_6_ (Tm^3+^) (ET1). The UEC luminescence colors of the SABTb-1, SABTb-2, SABTb-3 and SABTb-4 glass ceramics are characterized by the CIE chromaticity diagram, and the results are plotted in Fig. 5(b) ^37^. The luminescence color changes from blue (SABTb-1), to wathet blue (SABTb-2), then to white (SABTb-3), and finally to green (SABTb-4). The decay curves of the ^1^G_4_ state obtained by monitoring the emission of Tm^3+^ ions at 476 nm in the SABTb glass-ceramics are depicted in Fig. 5(c). The decay curves are approximately fitted with the single-exponential relationship, and the characteristic times are 677.36 μs, 656.74 μs, 605.13 μs and 580.97 μs for SABTb-1, SABTb-2, SABTb-3 and SABTb-4 glass ceramics respectively. Figure 5(d) shows the decay lifetime measurement results of ^1^G_4_ (Tm^3+^) and ^3^F_2,3_ (Tm^3+^). It can be seen that the energy transfer from Tm^3+^ to Tb^3+^ is indeed existent. That is to say, by adjusting the concentration of Tb^3+^ ions and changing the emission ratio of RGB, color tunable emitting can be achieved in this experiment.

**Figure 6:**
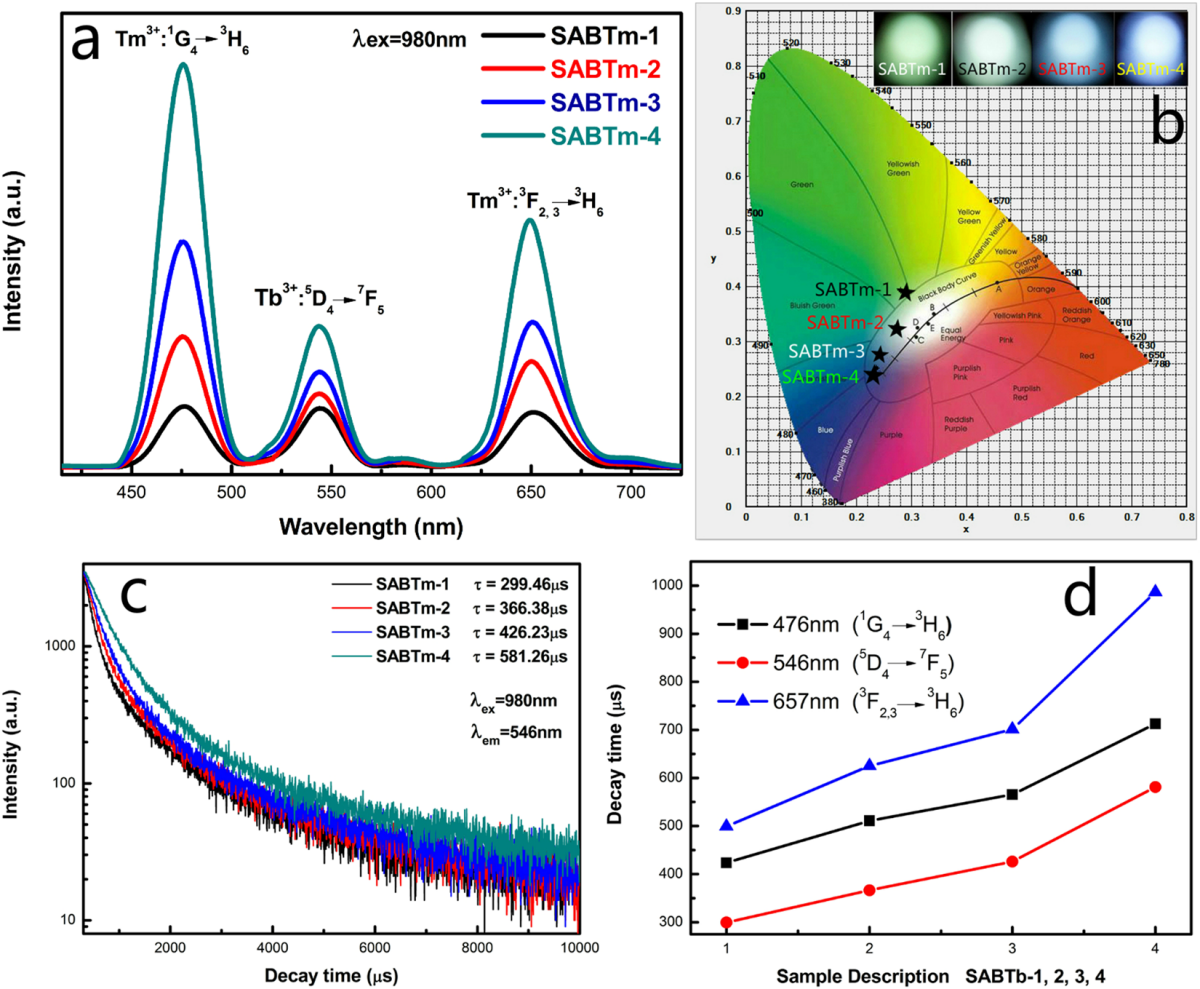
(**a**) The UEC emission spectra of SABTm-1, SABTm-2, SABTm-3 and SABTm-4 glass-ceramics. (**b**) CIE (X, Y) coordinate diagram showing chromaticity points of Tb^3+^ and Tm^3+^ luminescence in the glass-ceramics. (**c**) Fluorescence decay curves of Tb^3+^ ions at 546 nm in the SABTm glass-ceramics under 980 nm excitation. (**d**) Decay behavior of ^5^D_4_, ^1^G_4_ and ^3^F_2,3_ levels for the SABTm glass-ceramics.

Under constant concentration of Tb^3+^ ions in the glass composition, the effects of concentration variation of Tm^3+^ ions were also given for comparison in the second component of the SABTm-1, SABTm-2, SABTm-3 and SABTm-4 glass ceramics. Figure 6 shows the UEC emission spectra and decay time curves of the SABTm-1, SABTm-2, SABTm-3 and SABTm-4 glass ceramics heat-treated at 640 °C. As shown in Fig. 6(a), the UEC emission bands at 476 nm (Tm^3+^: ^1^G_4_ → ^3^H_6_) and 657 nm (Tm^3+^: ^3^F_2,3_ → ^3^H_6_) are enhanced dramatically with increasing concentration of Tm^3+^ ions in the SABTm glass-ceramics, and that at around 546 nm originated from Tb^3+^ ions also increases. These results imply that energy transfer from Tm^3+^ to Tb^3+^ ions may occur during the UEC process. The mechanism of energy transfer from Tm^3+^ to Tb^3+^ ions is suggested as follows: ^1^G_4_ (Tm^3+^) + ^7^F_5_ (Tb^3+^) → ^5^D_4_ (Tb^3+^) + ^3^H_6_ (Tm^3+^) (ET1). Figure 6(b) shows the calculated color coordinates according to the standard CIE chromaticity that is characterized by CIE chromaticity diagram^38^. As can be seen, the luminescence color changes from green (SABTm-1), to white (SABTm-2), then to wathet blue (SABTb-3), and finally to blue (SABTm-4)^36^. The UEC luminescence decay curves for ^5^D_4_ → ^7^F_5_ transition (546 nm) of Tb^3+^ in the SABTm glass were measured and illustrated in Fig. 6(c). Here, only approximate single-exponential luminescence decay curves are obtained, and the average lifetimes of ^5^D_4_ state are about 299.46 μs, 366.38 μs, 426.23 μs and 581.26 μs for SABTm-1, SABTm-2, SABTm-3 and SABTm-4 glass-ceramics respectively^39^. It is noticed that the lifetime of ^5^D_4_ state becomes longer, so that it can be proved that the energy transfer from Tm^3+^ to Tb^3+^ is indeed existent. Figure 6(d) shows the variation trends of lifetimes for the ^1^G_4_ (Tm^3+^), ^5^D_4_ (Tb^3+^) and ^3^F_2,3_ (Tm^3+^) states. Thus, we achieve the color tunability from green to white and blue emitting.

**Figure 7:**
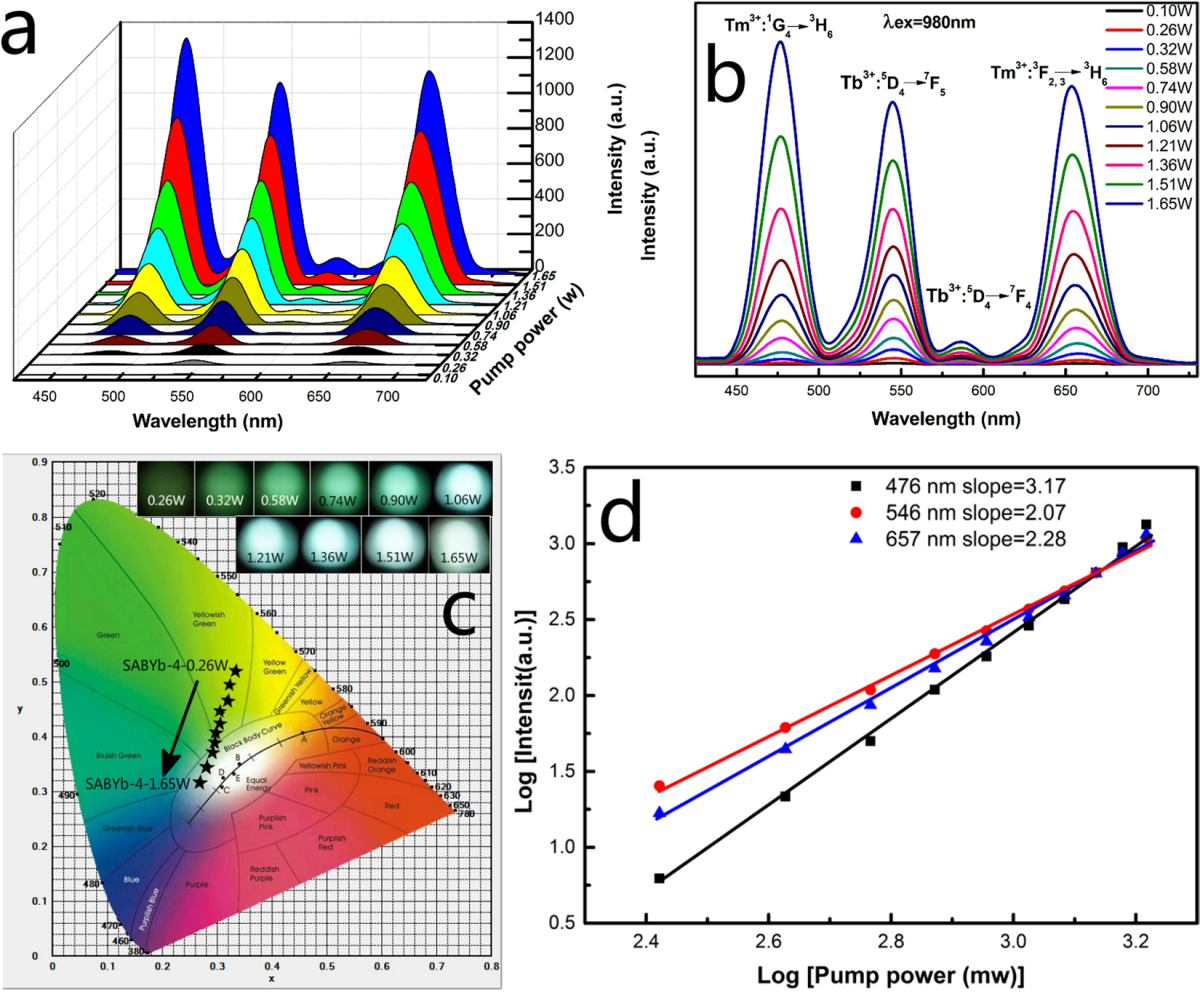
(**a** and **b**) The UEC emission spectra of the SABTm-1 glass-ceramics under adjustable power excitation of 980 nm. (**c**) CIE (X, Y) coordinate diagram showing chromaticity points of Tb^3+^ and Tm^3+^ luminescence in the glass-ceramics samples. (**d**) Log–log plots of the UEC emission intensity vs. the excitation power for the SABTm-1 glass-ceramics.

The color of bright emission from SAB glasses can be tuned easily by adjusting the exaction power of laser in this study. As shown in Fig. 7(c), the emission color of SABTm-1-640 glass-ceramics changes from yellow-green to white when the exaction power of laser increases from 0.26 to 1.65 W, which may be ascribed to the variation of the intensity ratio of RGB luminescences. As shown in Fig. 7(a) and (b), the intensity increasing rate of blue luminescence at 476 nm is higher than those of green luminescence at 546 nm and red luminescence at 657 nm when the exaction power of laser is increased. Therefore, it may be inferred that the UEC luminescences of blue, green and red color may involve multiphoton process with different exaction-steps. As far as multiphoton processes concerned, the relationship between the pumping power and the fluorescent intensity is I ∝ P^n40^, where I is the integrated intensity of the UEC luminescences (the integrated area of the UEC luminescence region), P is the pumping power of the excitation laser, and n is the photon number. The logarithmic transformation of the pumping power and fluorescence intensity is plotted in Fig. 7(d). The slopes of the logarithmic fitted lines for blue (476 nm), green (546 nm) and red (657 nm) luminescences are 3.17, 2.07 and 2.28, respectively. These results suggest that three-photon excitation is predominated in the conversion of 980 nm radiation into blue luminescence emission, whereas the green and red luminescences mainly come from the two-photon process^40^. Therefore, under focused laser irradiations with variable power, the intensity ratio of blue and green (or red) luminescences can be changed due to different multi-photon absorption steps of these UEC luminescences.

**Figure 8:**
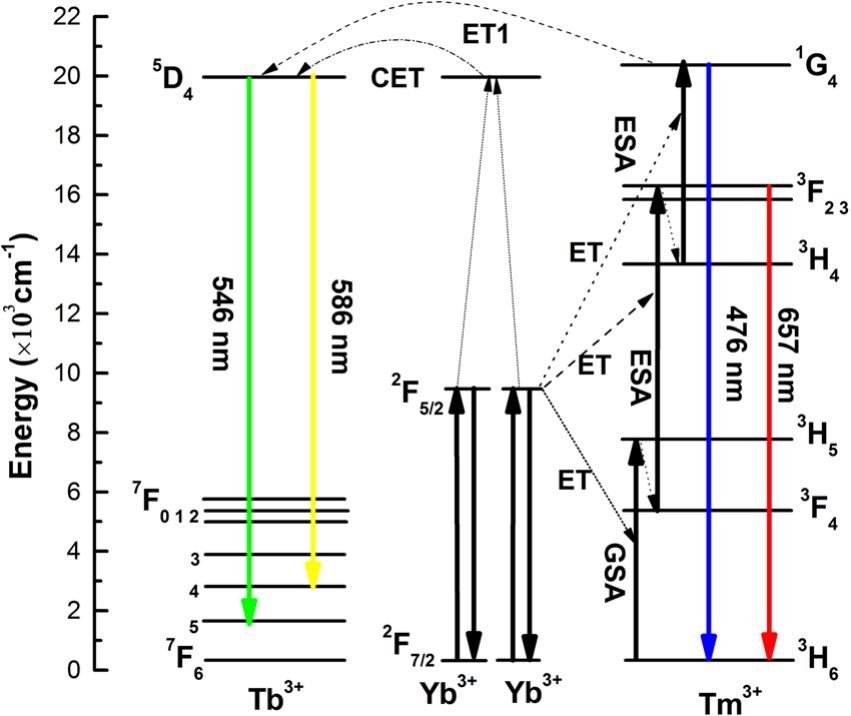
Mechanism for UEC and energy transfer processes of Tm^3+^/Tb^3+^/Yb^3+^ co-doped in the SAB glass-ceramics under 980 nm excitation.

The UC mechanism in the Tb^3+^/Tm^3+^/Yb^3+^ co-doped glass-ceramics are schematically depicted in Fig. 8. Firstly, Yb^3+^ ions are excited by the 980 nm laser diode radiation, which corresponds to Yb^3+^ ions: ^2^F_7/2_ → ^2^F_5/2_. Then, the energy transfers between Yb^3+^ ions and Tm^3+^ ions occur with considerably high efficiency, including the following pair of transitions as Yb^3+^ ions: ^2^F_5/2_ → ^2^F_7/2_ and Tm^3+^ ions: ^3^H_6_ → ^3^H_5_. Thirdly, the ^3^H_5_ excited state relaxes quickly to the metastate level ^3^F_4_ with the help of phonon relaxation. Fourthly, Yb^3+^ ions in the ^3^F_4_ state absorb a second photon of 980 nm or other Yb^3+^ ions in the ^2^F_5/2_ state transfer energy to the same Tm^3+^ ions, where the Tm^3+^ ions in the excited ^3^F_4_ state probably absorb a photon of 980 nm. After the excited state absorption (ESA), Tm^3+^ ions reach the ^3^F_2,3_ levels and then quickly relax to the ^3^H_4_ level with multi-phonon relaxing process. The population of ^1^G_4_ is based on the processes as follows: energy transfer from Yb^3+^ ions: ^2^F_5/2_ (Yb^3+^) + ^3^H_4_ (Tm^3+^) → ^1^G_4_ (Tm^3+^) + ^2^F_7/2_ (Yb^3+^) and ESA: ^3^H_4_ (Tm^3+^) + a photon → ^1^G_4_ (Tm^3+^). From the ^1^G_4_ level, Tm^3+^ ions decay to the ^3^H_6_ ground state, thus generating the intense blue emission at around 476 nm. Therefore, it is reasonable to deduce that the blue emission is a three-photon absorption process. The major contribution to the red (657 nm) emission is attributed to the ^3^F_2,3_ → ^3^H_6_ transition, so that the red emission is a two-photon absorption process. At the same time, the population of Tb^3+^ ions in the ^5^D_4_ excited-state level can be produced through the CET process of a pair of Yb^3+^ donor ions and a Tb^3+^ acceptor ion as follows^33, 34^: ^2^F_5/2_ (Yb^3+^) + ^2^F_5/2_ (Yb^3+^) + ^7^F_6_ (Tb^3+^) → ^5^D_4_ (Tb^3+^) + ^2^F_7/2_ (Yb^3+^) + ^2^F_7/2_ (Yb^3+^), resulting in the Tb^3+^ ions: ^5^D_4_ → ^7^F_J_ (J = 4, 5) radiative transitions at around 584 nm and 546 nm^41, 42^. Hence, the green emission is also a two-photon absorption process.

## Conclusions

In summary, we have demonstrated the highly efficient red, green and blue UEC luminescences in the Tb^3+^/Tm^3+^/Yb^3+^ co-doped SAB glass-ceramics based on multiphoton excitation in this paper. The UEC luminescences of Tb^3+^/Tm^3+^/Yb^3+^ co-doped SAB glass-ceramics were significantly enhanced in comparison with those of precursor glasses before heat treatment, and the RGB ratios of UEC luminescences and the decay times of these glass-ceramics could be tuned by changing the concentration of doped RE ions and adjusting the laser power simultaneously. The blue and red UEC luminescences of Tm^3+^ were found to originate from three-photon and two-photon excitations respectively, while the green UEC luminescence of Tb^3+^ was from a two-photon excitation. In addition, it was also proved that energy transfers during the UEC process included the transfers from Yb^3+^ to Tm^3+^ and Tb^3+^, as well as the transfer from Tm^3+^ to Tb^3+^. Our work suggests a possible route to design and develop the red, green and blue UEC luminescence materials by laser, and provides useful information for further development of UEC glass-ceramics associated with the energy transfer between Tm^3+^ and Tb^3+^ ions.

## Electronic supplementary material

Supplementary information
