# Supplementary material for: Adjustable multicolor up-energy conversion in light-luminesce in Tb3+/Tm3+/Yb3+ co-doped oxyfluorifFde glass-ceramics containing Ba2LaF7 nanocrystals
Source: Sci Rep. 2017 Jul 26;7:6518. doi: 10.1038/s41598-017-05943-4 (PMC5529515; doi:10.1038/s41598-017-05943-4)
Supplement: Supplementary file 1 — Supplementary information [file 41598_2017_5943_MOESM1_ESM.pdf]

Supplementary Information

Adjustable multicolor up-energy conversion in light-luminesce in $Tb^{3+}/Tm^{3+}/Yb^{3+}$ co-doped oxyfluoride glass-ceramics containing Ba_2LaF_7 nanocrystals

Zhencai Li [1], Dacheng Zhou [1, 2]*, Yong Yang [1, 2], Peng Ren [1], Jianbei Qiu [1, 2]*

[a] Faculty of Material Science and Engineering, Kunming University of Science and Technology, Kunming 650093, China

[b] Key Lab. of Advanced Materials of Yunnan Province, Kunming 650093, China

Materials. SiO_2 (99.99%), Al_2O_3 (99.99%), Na_2CO_3 (99.99%), BaF_2 (99.99%), LaF_3 (99.99%), TbF_3 (99.99%), TmF_3 (99.99%) and YbF_3 (99.99%) were all purchased from Aladdin and used as starting materials without further purification.

Characterization. DSC analysis was carry out on a NETZSCH STA 449F3 STA449F3A-0413-M. XRD analysis was carried out on a TD-3500 X-ray diffractometer with Cu Kα radiation(λ = 1.5406 Å).The luminescence spectra were obtained with a HITACHI F-7000 fluorescence spectrophotometer equipped with a R928 photo counting photoplier tube (PMT), in conjunction with a 980 nm diode laser. Photographs were taken with a Canon PowerShot G9 digital camera.

Glass compositions. The specify x, y and z-values in the glass compositions. The purpose of our experimental is to change the amount of different doping can achieve color changes can also be achieved on the white light. The most difficult to determine the concentration of Tm^{3+}, because the higher the concentration of Tm^{3+}, the up-conversion of blue light is faster than that of red light emission. Therefore, the low

concentration of Tm^{3+} can reduce the intensity of blue emission, which is almost the same as that of red light emission. Again, I references: Upconversion Luminescence with Adjustable MultiColor in Rare Earth Co-Doped Transparent Oxyfluoride Glasses. Finally, the concentration of Tm^{3+} and Yb^{3+}were determined by comparing with our experiments.

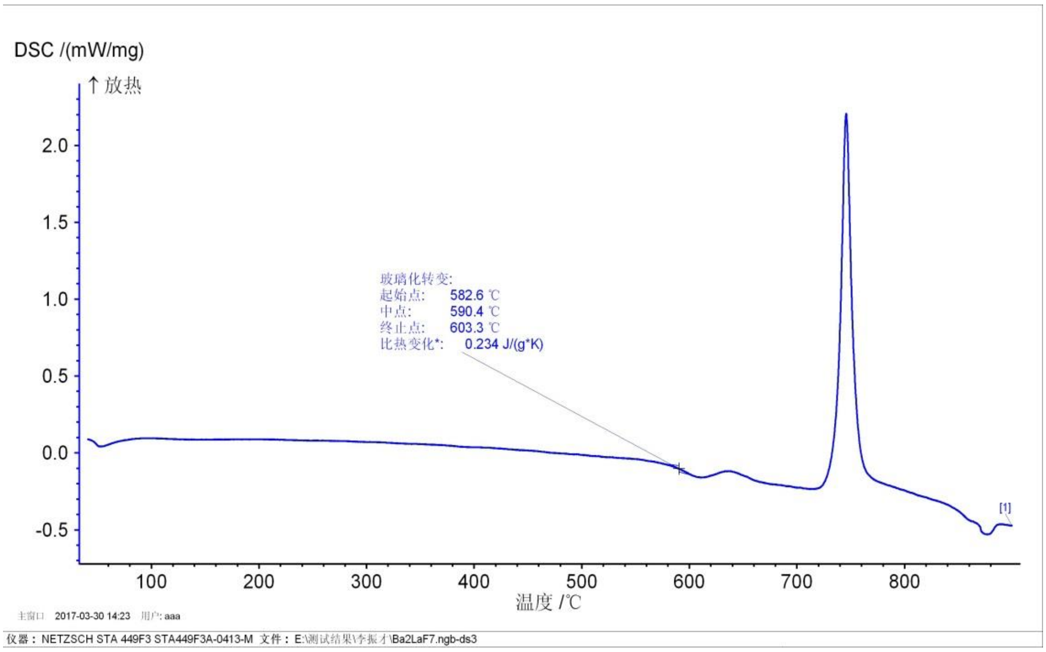

Figure 1. The DSC curve of the SABYb-2 glass sample.

The transition temperature should be the midpoint of the temperature of 590 ℃, but I mean the starting point of the glass transition temperature of 583 ℃. For the glass transition temperature, the glass transition temperature midpoint temperature is the most valuable reference.

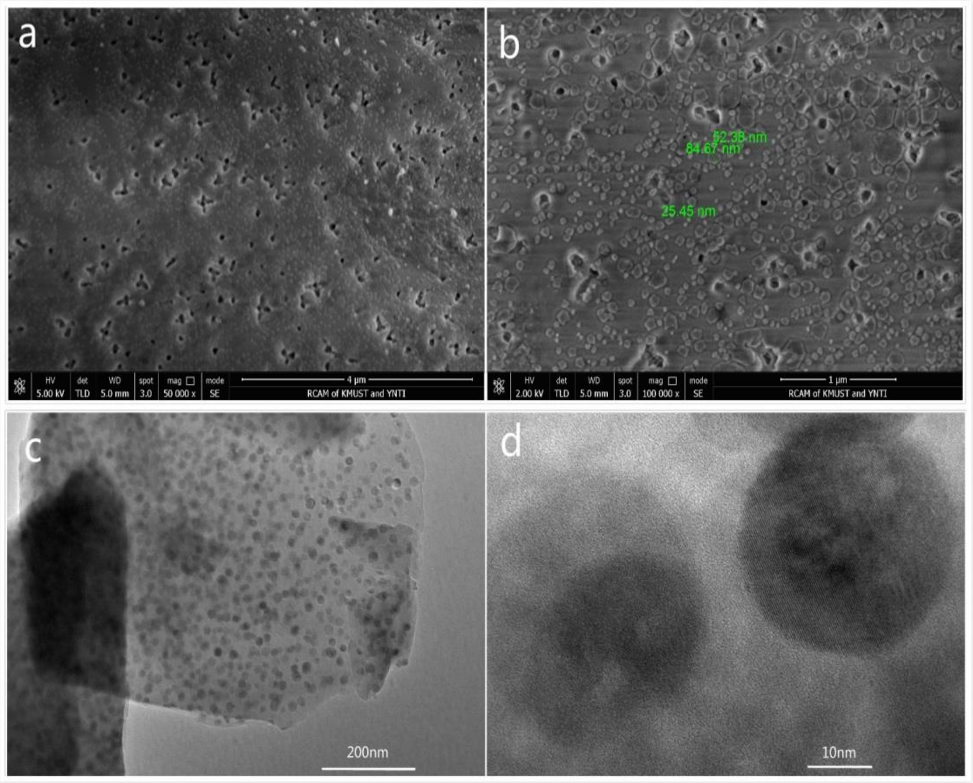

Figure 2(a and b). The SEM images of the SABYb-2-640 glass-ceramics after heat-treated at 640 ℃ for 2 h. (c) TEM micrograph of SABYb-2 glass-ceramics after heat-treated at 640 ℃ for 2 h. (d) High resolution transmission electron microscope (HRTEM) image of SABYb-2 glass-ceramics.

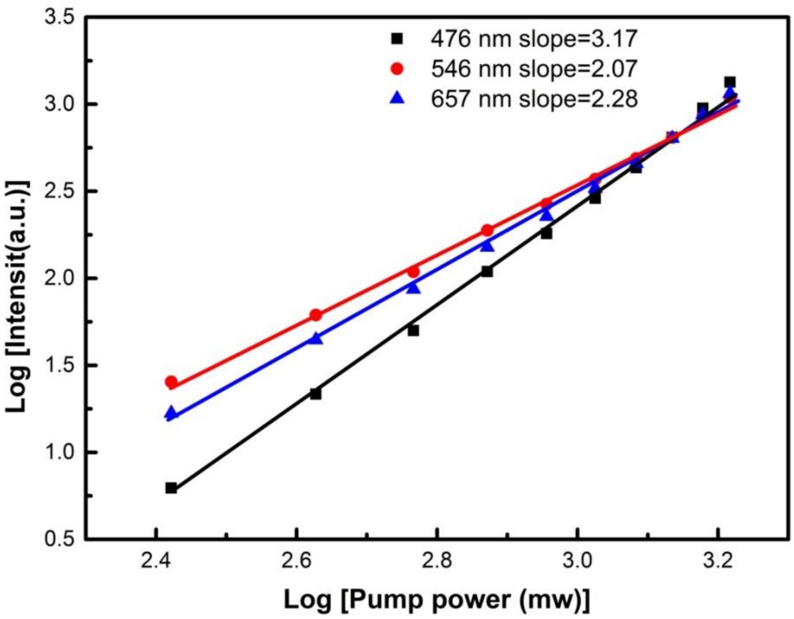

Figure 7(d). Log–log plots of the UEC emission intensity vs. the excitation power for the SABTm-1 glass-ceramics. The following figure shows the original data.

As far as multiphoton processes concerned, the relationship between the pumping power and the fluorescent intensity is $I \propto P^n$, where I is the integrated intensity of the UEC luminescences (the integrated area of the UEC luminescence region), P is the pumping power of the excitation laser, and n is the photon number. The logarithmic transformation of the pumping power and fluorescence intensity is plotted in Fig. 7(d). The slopes of the logarithmic fitted lines for blue (476 nm), green (546 nm) and red (657 nm) luminescences are 3.17, 2.07 and 2.28, respectively. These results suggest that three-photon excitation is predominated in the conversion of 980 nm radiation into blue luminescence emission, whereas the green and red luminescences mainly come from the two-photon process.
